# Hearing impairments caused by genetic and environmental factors

**DOI:** 10.1007/s12199-012-0300-z

**Published:** 2012-08-17

**Authors:** Nobutaka Ohgami, Machiko Iida, Ichiro Yajima, Haruka Tamura, Kyoko Ohgami, Masashi Kato

**Affiliations:** Unit of Environmental Health Sciences, Department of Biomedical Sciences, College of Life and Health Sciences, Chubu University, No. 50 Building 11 floor, 1200 Matsumoto, Kasugai, Aichi 487-8501 Japan

**Keywords:** Hearing loss, c-Ret, Ednrb, Spiral ganglion neuron, Neurodegeneration, Balance, Noise

## Abstract

Impairments of hearing and balance are major problems in the field of occupational and environmental health. Such impairments have previously been reported to be caused by genetic and environmental factors. However, their mechanisms have not been fully clarified. On the other hand, the inner ear contains spiral ganglion neurons (SGNs) in the organ of Corti, which serve as the primary carriers of auditory information from sensory cells to the auditory cortex in the cerebrum. Inner ears also contain a vestibule in the vicinity of the organ of Corti—one of the organs responsible for balance. Thus, inner ears could be a good target to clarify the pathogeneses of sensorineural hearing losses and impaired balance. In our previous studies with *c*-*Ret* knock-in mice and *Endothelin receptor B* (*Ednrb*) knock-out mice, it was found that syndromic hearing losses involved postnatal neurodegeneration of SGNs caused by impairments of *c*-*Ret* and *Ednrb*, which play important roles in neuronal development and maintenance of the enteric nervous system. The organ of Corti and the vestibule in inner ears also suffer from degeneration caused by environmental stresses including noise and heavy metals, resulting in impairments of hearing and balance. In this review, we introduce impairments of hearing and balance caused by genetic and environmental factors and focus on impairments of SGNs and the vestibule in inner ears as the pathogeneses caused by these factors.

## Introduction

It has been reported that about 250 million people worldwide suffer from hearing losses. About 30 % of people with congenital hearing loss are syndromic and the remaining 70 % are non-syndromic. In addition, most elderly people develop age-related (late-onset) hearing loss [1–3]. In general, these hearing losses have been classified as different diseases due to distinct pathogeneses [1, 2]. Sensorineural hearing losses are caused by impairments of inner ears and are difficult to cure due to the location and complex morphology of inner ears [1, 2]. Sensorineural hearing loss is a clinically heterogeneous disease leading to negative impacts on quality of life (QOL) in all generations. Sensorineural hearing loss involves different onset, severity and pathological sites.

Inner ears have been analyzed in order to clarify the pathogeneses of sensorineural hearing losses. The inner ears contain the organ of Corti and stria vascularis (SV). The SV is essential for maintenance of endolymph potential. The organ of Corti contains two kinds of sensory cells [inner hair cells (IHCs) and outer hair cells (OHCs)] and plays an important role in mechanotransduction, by which sound stimuli are converted into electric stimuli. Auditory information from the sensory cells is transferred to spiral ganglion neurons (SGNs) as the primary carriers and is eventually transferred to the auditory cortex in the cerebrum [1, 2]. The SV consists of marginal cells, melanocytes (also known as intermediate cells) and basal cells, and has been shown to maintain high levels of potassium ion for endocochlear potential (EP) [4, 5]. Melanocytes in the inner ear are located specifically in the SV, and defects in melanocytes lead to impaired EP levels resulting in hearing loss [6]. Thus, disturbance of these constituent cells in inner ears has been shown to cause hearing losses [7]. Inner ears also contain a vestibule in the vicinity of the organ of Corti. Vestibular hair cells covered with otoconia play an important role in mechanotransduction, by which gravity impulses are converted into neural impulses. Impairments of vestibular hair cells have been shown to cause abnormal behaviors including balance [8]. Thus, the vestibule containing hair cells and an otolith is one of the organs responsible for balance.

Impairments of hearing and balance—both major problems in the field of occupational and environmental health—are caused by the intricate interplay of genetic, aging and environmental factors [1–3]. However, there is limited information about the pathogenesis of hearing loss and imbalance. This review focuses on hearing impairments caused by neurodegeneration of SGNs due to impairments of hearing-related genes (c-*Ret* and *Ednrb*) and by environmental stresses [low frequency noise (LFN) and heavy metals].

## c-Ret-mediated hearing losses

c-RET is a receptor-tyrosine kinase [9]. Glial cell line-derived neurotrophic factor (GDNF)—one of the ligands for c-RET—exerts its effect on target cells by binding to a glycosyl phosphatidylinositol (GPI)-anchored cell surface protein (GFRα1). This binding facilitates the formation of a complex with the receptor tyrosine kinase c-RET. Formation of this complex activates c-RET autophosphorylation as a trigger for c-RET-mediated signaling pathways to give positive signals for cell survival [9–12]. Previous studies have also indicated that GDNF stimulates a Ret-independent signaling pathway [10, 13, 14]. Tyrosine 1062 (Y1062) in c-Ret plays an important role in kinase activation as one of the autophosphorylation sites, and is also a multi-docking site for several signaling molecules including SHC, a transmitter for c-Ret-mediated signaling pathways [13, 15, 16]. In both mice and humans, c-*RET* has been shown to be essential for the development and maintenance of the enteric nervous system (ENS) [13, 15] and to be the most frequent causal gene of Hirschsprung disease (HSCR; megacolon disease) (in 20–25 % of cases) in humans [17, 18]. In fact, severe HSCR (e.g., total intestinal agangliosis and impaired development of the kidney) has been shown to develop in homozygous knock-in mice in which Y1062 in c-Ret was replaced with phenylalanine (*c*-*Ret*-KI^Y1062F/Y1062F^-mice), while heterozygous c-Ret Y1062F knock-in mice (*c*-*Ret*-KI^Y1062F/+^-mice) are reported to have no HSCR-linked phenotypes [11]. Thus, the results of previous studies indicate that HSCR in mice develops recessively [11], while HSCR in humans has been shown to develop dominantly due to RET mutations [19]. As described above, *c*-*Ret* and *c*-*RET* are crucial genes for HSCR; however, there had been no direct evidence to link *c*-*Ret* and *c*-*RET* to hearing impairments in mice or humans. Our recent studies have shown that complete unphosphorylated Y1062 in c-Ret, with no change in expression level, caused congenital hearing loss in *c*-*Ret*-KI^Y1062F/Y1062F^-mice [20], while partially unphosphorylated c-Ret led to normal hearing development until 1 month of age but then accelerated age-related hearing loss in *c*-*Ret*-KI^Y1062F/+^-mice [21]. Thus, impairments of *c*-*Ret* phosphorylation monogenetically result in early-onset syndromic hearing loss as well as late-onset non-syndromic hearing loss. Our results correspond in part to the results of previous studies demonstrating that c-Ret, GFRα1 and GDNF are expressed in auditory neurons [22, 23] and that GDNF has a protective effect on antibiotic-mediated ototoxicities [24–27].

## Ednrb-mediated hearing loss

**Fig. 1:**
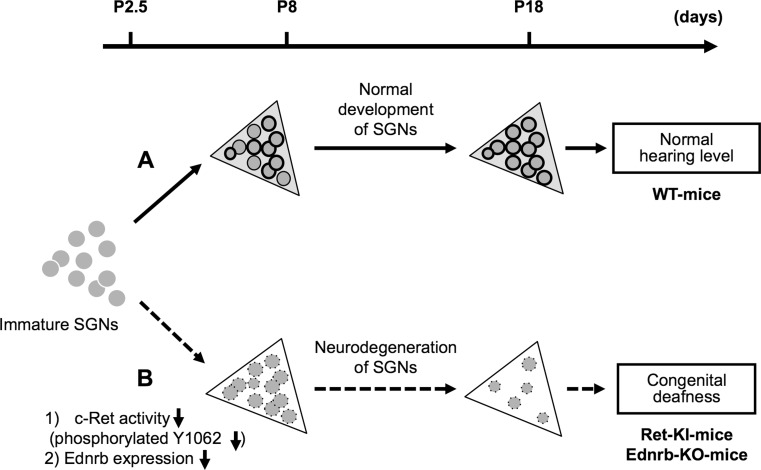
Schematic summary of congenital deafness caused by neurodegeneration of spiral ganglion neurons (SGNs) in *c*-*Ret*-knock-in-mice and *Ednrb*-knock-out-mice. The *x*-axis indicates age (days after birth) of mice. *Triangles* Rosenthal’s canals in wild-type (WT) (*light gray background*), or homozygous *c*-*Ret*-knock-in^Y1062F/Y1062F^ (Ret-KI) [20] and homozygous *Ednrb*-knock-out-mice (Ednrb-KO) (*white background*) [36]; *gray circles/no outline* immature SGNs; *gray circles/thin outline* SGNs;* gray circles/bold outline* SGNs with “phosphorylated Y1062 in c-Ret” or “expression of Ednrb”. *Dark gray circles/dotted outline* SGNs with “decreased phosphorylation of Y1062 in c-Ret” or “decreased expression of Ednrb”. **a** *c*-*Ret*-KI- and *Ednrb*-KO-mice suffer from congenital deafness with neurodegeneration of SGNs. **b** *c*-*Ret*-KI^Y1062F/Y1062F^-mice showed no Y1062-phosphorylated SGNs even on P8, although Y1062-phosphorylated SGNs began to appear in WT mice from P8 [20]. *Ednrb*-KO-mice also showed undetectably low expression level of Ednrb in SGNs on P8, although Ednrb-positive SGNs began to appear in WT mice from P8 [36]

Waardenburg-Shah syndrome (WS type IV, WS-IV), which is caused by mutations in the transcription factor Sox10 [28], cytokine endothelin (ET)-3 [29] and its receptor endothelin receptor B (Ednrb) [30], is characterized by hypopigmentation, megacolon disease and hearing loss. The incidence of WS is 1 per 10,000 to 20,000 people [31]. Endothelin receptor B (Ednrb/EDNRB) belongs to the G-protein-coupled receptor family that mediates the multifaceted actions of endothelins [32, 33]. Mutations of *Ednrb/EDNRB* have been shown to cause embryonic defects in melanocytes and enteric ganglion neurons derived from the neural crest, resulting in hypopigmentation, megacolon disease and congenital hearing loss. In previous studies with animal models, both piebald-lethal rats in which *Ednrb* is spontaneously mutated [34] and Ednrb homozygous knock-out [*Ednrb*(−*/*−)] mice [35] have been shown to have typical WS-IV phenotypes. Thus, previous studies indicate that Ednrb is a key regulatory molecule for embryonic development of melanocytes and peripheral neurons, including neurons in the ENS. Previous studies also demonstrated that impairments of *Ednrb/EDNRB* cause syndromic hearing loss due to congenital defects of melanocytes in the stria vascularis of the inner ear [30, 32–35]. In our previous study, Ednrb protein was expressed in SGNs from wild-type (WT)-mice on postnatal day 19 (P19), while it was undetectable in SGNs from WT-mice on P3. Correspondingly, *Ednrb* homozygously deleted mice [*Ednrb*(−*/*−)-mice] developed congenital hearing loss (Fig. 1) [36]. Thus, expression of Ednrb expressed in SGNs in the inner ears is required for postnatal development of hearing in mice. A therapeutic strategy for congenital hearing loss in WS-IV patients has not been established. EDNRB expressed in SGNs could be a novel potential therapeutic strategy for congenital hearing loss in WS-IV patients.

## Neurodegeneration of SGNs caused by impairments of c-Ret and Ednrb

Phosphorylation of Y1062 in c-Ret has been shown to mediate several biological responses, including development and survival of neuronal cells [13, 37]. In our recent studies, *c*-*Ret*-KI^Y1062F/Y1062F^-mice developed severe congenital deafness with neurodegeneration of SGNs on postnatal day (P) 8-18, while *c*-*Ret*-KI^Y1062F/Y1062F^-mice showed morphology of SGNs comparable to that in WT mice on P2-3 [20]. Phoshorylation of Y1062 in c-Ret of SGNs from WT mice on P2-3 was below the limit of detection, while that on P8-18 was clearly detectable [20]. Thus, it is thought that SGNs from *c*-*Ret*-KI^Y1062F/Y1062F^-mice developed normally at least until P3 after birth, when Y1062 in c-Ret of SGNs from WT mice is unphospholylated. However, in *c*-*Ret*-KI^Y1062F/Y1062F^-mice, phosphorylation of Y1062 is no longer maintained by P8–P18, when Y1062 in c-Ret of SGNs from WT mice exhibits significant phosphorylation [20]. Furthermore, partially unphosphorylated Y1062 in c-Ret of SGNs accelerated age-related hearing loss with accelerated reduction of SGNs from 4 months of age, while normal hearing and normal density of SGNs were observed at least until 1 month of age, when hearing has matured [21]. On the other hand, Ednrb protein was expressed in SGNs from WT-mice on postnatal day 19 (P19), while it was undetectable in SGNs from WT-mice on P3. Correspondingly, *Ednrb*(−*/*−)-mice with congenital hearing loss showed a decreased number of SGNs (Fig. 1) and degeneration of SGNs on P19 but not on P3 [36]. Thus, our results show that Ednrb expression in SGNs in inner ears is required for postnatal survival of SGNs in mice. The neurodegeneration of SGNs from *c*-*Ret*-KI^Y1062F/Y1062F^-mice and *Ednrb*(−*/*−)-mice did not show typical apoptotic signals and did not involve disturbance of hair bundles of IHCs and OHCs [20, 36]. The congenital hearing loss involving neurodegeneration of SGNs as well as megacolon disease in *Ednrb*(−*/*−)-mice were improved markedly by introducing an *Ednrb* transgene under the control of the dopamine beta-hydroxylase promoter (*Ednrb*(−*/*−); *DBH*-*Ednrb*-mice). Neurodegeneration of SGNs was restored by introducing constitutively activated RET also in the case of c-Ret-mediated hearing loss. Thus, our results indicate that c-RET and EDNRB expressed in SGNs could be molecular targets in the prevention of hearing impairments.

## Environmental stress-related impairments of hearing and balance

**Fig. 2:**
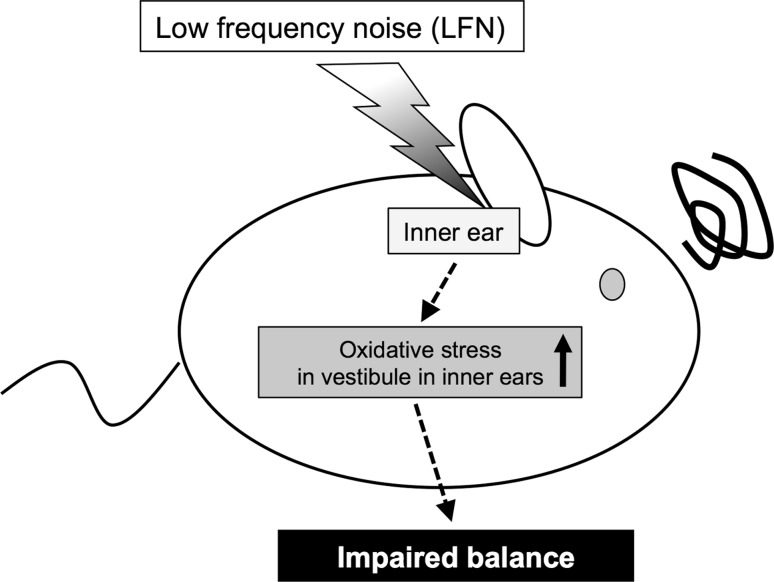
Schematic summary of impaired balance in mice caused by exposure to low frequency noise (LFN). Chronic exposure to low frequency noise (LFN, 0.1 kHz) at moderate levels of 70 dB sound pressure level (SPL) causes impaired balance involving morphological impairments of the vestibule with enhanced levels of oxidative stress [39]

Exposure to noise is recognized as one of the major environmental factors causing hearing loss [1]. Noise consists of sound with broad frequencies, but there is limited information about the frequency-dependent influence of noise on health. Low frequency noise (LFN) is constantly generated from natural and artificial sources. The frequency range of LFN is usually defined as being below 100 Hz, while that of infrasound is usually below 20 Hz [38]. In our recent study, we found that chronic exposure to LFN at moderate levels of 70 dB sound pressure level (SPL) causes impaired balance involving morphological abnormalities of the vestibule with increased levels of oxidative stress (Fig. 2) [39]. Previous studies have shown that behavioral impairments induced by antibiotics involved degeneration of vestibular cells and oxidative stress [40, 41]. In addition, a previous study has shown that antioxidant compounds prevent noise-induced hearing loss [42]. Ototoxicity caused by oxidative stress in inner ears has been shown to accompany impairment of antioxidant enzymes [42]. Thus, existing studies indicate the necessity for further investigation of a causal molecule related to oxidative stress in vestibular hair cells affected by LFN, and of the preventive effect of antioxidants on impaired balance caused by LFN exposure. On the other hand, exposure to heavy metals including mercury, cadmium and arsenic has been suggested to cause impairments in balance [43] and hearing [44–46] in humans and experimental animals. Smoking has also been shown to affect hearing in humans [47]. In previous studies, childhood exposure to heavy metals has been shown to sensitively affect hearing development in humans [48–50]. Aging has also been shown to affect sensitivities to ototoxic factors in mice [51]. Therefore, further studies are needed to determine the age-specific susceptibilities to environmental stresses, including heavy metals, in terms of ototoxicity in mice and humans.

## Conclusions

Our studies provide direct evidence that c-RET and EDNRB expressed in SGNs are novel targets for hearing loss. These studies underline the importance of considering the activity as well as the expression of the target molecule in order to elucidate the etiologies of hereditary deafness. In addition, environmental stresses, including exposure to noise and heavy metals, can cause impairments of hearing and balance that are affected intricately by aging and genetic factors. Information obtained in previous studies prompts further investigation of the influence of environmental stresses on the impairment of hearing and balance with consideration of aging and genetic factors to develop new diagnostic, preventive and therapeutic strategies against impairment of hearing and balance.
